# Development of synthetic nuclear melt glass for forensic analysis

**DOI:** 10.1007/s10967-015-3941-8

**Published:** 2015-01-20

**Authors:** Joshua J. Molgaard, John D. Auxier, Andrew V. Giminaro, C. J. Oldham, Matthew T. Cook, Stephen A. Young, Howard L. Hall

**Affiliations:** 1 Department of Nuclear Engineering, University of Tennessee, Knoxville, TN 37996 USA; 2 Radiochemistry Center of Excellence (RCoE), University of Tennessee, Knoxville, TN 37996 USA; 3 Institute for Nuclear Security, University of Tennessee, Knoxville, TN 37996 USA; 4 Engineering Science and Mechanics, University of Tennessee, Knoxville, TN 37996 USA; 5 Department of Physics and Nuclear Engineering, United States Military Academy, West Point, NY 10996 USA

**Keywords:** Debris, Nuclear weapons, Nuclear forensics, Trinitite, Melt glass, Morphology

## Abstract

A method for producing synthetic debris similar to the melt glass produced by nuclear surface testing is demonstrated. Melt glass from the first nuclear weapon test (commonly referred to as trinitite) is used as the benchmark for this study. These surrogates can be used to simulate a variety of scenarios and will serve as a tool for developing and validating forensic analysis methods.

## Introduction

The illicit use of nuclear material is one of the major challenges of the modern era. The threat of nuclear proliferation and nuclear terrorism is a continued and growing concern [1]. This threat has been recognized by congress and was the primary motivation for the passage of the Nuclear Forensics and Attribution Act [2]. This act called for the development of a credible capability for identifying sources of nuclear material used in a terrorist act, and also acknowledged the challenge presented by the dwindling number of radiochemical programs and facilities in the United States.

The Radiochemistry Center of Excellence (RCoE) established at the University of Tennessee (UT) by the National Nuclear Security Administration (NNSA) seeks to partially fill the academic gap and meet the challenges identified in the Nuclear Forensics Attribution Act. One of the primary focus areas of the RCoE is the development of improved radiochemical separation and analysis methods with the goal of reducing the time required for accurate post-detonation analysis. Surrogate material that is accessible to the academic community is required to enable the development of appropriate forensic methods and to train future specialists in this field.

Nuclear waste vitrification studies have provided useful information regarding the immobilization of radioactive materials within oxide glass systems [3]. While thermodynamic stability and radiation tolerance are of primary importance for vitrified waste glasses [4], the key issue for synthetic representations of post-detonation nuclear melt glass is that the matrix be an accurate representation based on the estimated composition of the carrier material and simulated nuclear event parameters (e.g. fuel type, weapon yield, and emplacement scenario). These melt glasses are expected to be highly heterogeneous and contain numerous structural defects.

The process outlined here leads to the creation of synthetic nuclear melt glass similar to trinitite. This process will be optimized and extended to establish a capability for surrogate debris production. The ultimate goal is to provide the nuclear forensics community with a robust method to supply realistic surrogate materials simulating a variety of detonation scenarios. This work will also allow for the development of more analytical techniques for investigating post-detonation material, specifically the use of thermo-chromatography as a rapid separation method, currently under development at UT [5]. Methods involving high spatial resolution have been applied to the analysis of trinitite. In particular microscopic X-ray fluorescence (XRF) elemental mapping and backscatter electron (BSE) microscopy have been employed to highlight the heterogeneity observed in trinitite [6]. The voids observed at both the macroscopic and microscopic level are of particular interest as well as the spatial distribution of elements and compounds. While the use of high-resolution imaging techniques would likely have limited use in an actual forensics application, these techniques provide excellent methods for comparison of the synthetic trinitite formulation to actual trinitite. To support the use of such methods for future analysis the current study has focused on producing a melt glass surrogate with realistic physical, chemical, and morphological properties.

Previous melt glass production efforts have been focused on replicating the chemical and radioactive properties of particulate debris [7]. The work presented here emphasizes comparisons of crystalline morphology and microstructure between trinitite and synthetic melt glass. Specifically, the quartz content and the degree of amorphousness is quantified for both trinitite and synthetic melt glass, and the void content is also compared.

The process employed here is also unique in that it produces a bulk solid melt glass via rapid melting in a pre-heated furnace. The melts are produced in a single phase without mixing in order to preserve the defects and heterogeneity which is a notable feature of trinitite. The melts are also quenched in sand to simulate the rapid temperature transition experienced by surface melt glass following a nuclear detonation.

## Experimental

### Materials

Trinitite samples were purchased from the Mineralogical Research Company (http://www.minresco.com/), San Jose, CA. These samples were analyzed for comparison with the synthetic melt glass samples produced for this study and these will be referenced throughout this paper. Metals and metal oxide powders were purchased from Sigma-Aldrich, Saint Louis, MO, and from Fisher Scientific, Pittsburgh, PA. Uranyl nitrate hexahydrate (UNH) and potassium hydroxide where also purchased from Fisher Scientific. These reagents were used as received without further purification to develop powder formulations, which form glasses when melted at high temperature and then rapidly cooled.

### Preparation of synthetic debris matrices

Oxide mixtures were prepared in accordance with published data regarding trinitite composition [8]. The resulting matrix is known as the standard trinitite formulation (STF).

The STF was used as a starting point for the development of synthetic nuclear melt glass specifications. Individual oxide powders were carefully weighed and then thoroughly mixed using a mortar and pestle. KOH pellets and Na_2_O beads were powdered prior to fine mixing.

It is possible that oxygen, nitrogen dioxide, and water molecules from the atmosphere, along with carbon from the graphite crucibles, will react with other metals in the sample during melting and subsequent cooling to produce various oxide compounds. These products are not entirely undesirable from an experimental standpoint as the atmosphere surrounding a nuclear explosion will contain gases and volatilized organic matter.

The majority of the samples discussed in the results section of this paper do not contain uranium. The tamper was omitted to avoid potential challenges associated with handling of radioactive samples and because small quantities of uranium (or UNH) will not impact the final morphology of the samples (which is the primary concern of this present study). It is planned to incorporate uranium into future samples, which will then be exposed to a high neutron flux, thus generating both fission and activation products.

### Melt glass production

The heat generated by a nuclear explosion will produce temperatures as high as 8,430 °C [9] leaving most materials near ground zero in a plasma state. This environment cannot be easily recreated with standard equipment in a laboratory. However, it is believed that the critical parameters of nuclear melt glass formation are the soil solidification temperature and corresponding solidification time. The bulk of the melted and vaporized material pulled into the fireball will cool rapidly and reach its re-solidification temperature within a few seconds after the explosion. Partially molten droplets will fall to the earth and form a glassy layer on top of the fused sand [10].

The conditions which are replicated experimentally are those existing at the moment of soil solidification. This is accomplished using a Carbolite HTF 18/4 1800C Box Furnace rated at 1,800 °C.

Silicon dioxide (the most abundant compound in most soils) melts at approximately 1,600 °C. Oxide mixtures which also include aluminum oxide, calcium oxide, sodium oxide and potassium oxide will often melt at lower temperatures (between 1,200 and 1,400 °C) [3]. The liquidus temperature calculator developed by A. Fluegel and available at http://glassproperties.com/liquidus/ can be used to estimate the melting temperatures of six-component glass forming networks [11]. Table 1 shows a comparison between the STF and a typical silica glass. The composition of the modeled glass was chosen to be as close as possible to the STF while remaining within the validity limits of the model. The melting temperature of the six-oxide system is based on the disconnected peak function method [11]. Other modeling methods may be used to estimate liquidus temperatures [12, 13]. For purposes of this study a rough approximation is adequate since the processing temperature will be set well above the estimated melting temperature. Assuming the liquidus temperature is exceeded, the critical experimental parameter is the subsequent cooling rate and solidification time.

**Table 1:** Estimated STF melting temperature

Synthetic trinitite		Modeled glass^a^	
Compound	Wt %	Compound	Wt %
SiO_2_	64.21	SiO_2_	63.25
CaO	9.64	CaO	9.64
Na_2_O	1.25	Na_2_O	5.57
Al_2_O_3_	14.27	Al_2_O_3_	14.27
KOH	6.12	K_2_O	6.12
MgO	1.15	MgO	1.15
Liquidus temperature		1,275 °C	±38 °C

^a^ http://glassproperties.com/liquidus/

The Carbolite HTF is heated to a temperature at least 100 °C higher than the estimated melting temperature of the specified matrix. The powder sample, contained within a graphite crucible, is then introduced into the hot furnace. The sample is heated long enough to form a melt, usually in the form of a small bead. The crucible and sample are then removed from the furnace and the sample is immediately quenched at room temperature. Quenching is accomplished by pouring the molten bead directly onto a thick layer of cool quartz sand. Grains of sand which become fused to the glass bead can be removed by polishing. The glass cools to room temperature within 30–60 s.

Table 2 lists the processing parameters for the synthetic samples produced for this study, along with information about the trinitite samples. The trinitite sample names provided by the Mineralogical Research Company are listed in the source column. All synthetic samples were produced in the laboratory at UT by melting approximately two grams of the STF powder in a graphite crucible. The sample numbers will be used throughout this paper to reference and compare the trinitite and synthetic samples.

**Table 2:** Trinitite and synthetic sample data

No.	Matrix	Source	Temp (^ο^C)	Time (min)
S1	STF	Lab	1,400	60
S2	STF	Lab	1,400	45
S3	STF	Lab	1,500	60
S4	STF	Lab	1,500	45
T1	Trinitite	T1003	UNK	UNK
T2	Trinitite	T2016	UNK	UNK
T3	Trinitite	T2024	UNK	UNK
T4	Trinitite	T2025	UNK	UNK
T5	Trinitite	T2026	UNK	UNK
T6	Trinitite	T3001	UNK	UNK
T7	Trinitite	T5055	UNK	UNK
T8	Trinitite	T4new	UNK	UNK

### Characterization

Trinitite samples purchased from the Mineralogical Research Company were analyzed via powder X-ray diffraction (P-XRD), scanning electron microscopy (SEM), and energy dispersive spectroscopy (EDS). Synthetic samples were analyzed using the same techniques and compared to trinitite to examine chemical and morphological similarities.

P-XRD analysis was performed on trinitite and synthetic melt glass samples using a Panalytical Empyrean X-ray diffractometer with a Pixcel 3D detector. The X-ray source was a Cu anode set at 40 mA and 45 kV. A slit window of 1/4° 2*θ* was used along with a 1/8° 2*θ* anti-scatter diffraction grating. All samples were measured using a silicon (001) no-background sample holder and were set to spin at 4 revolutions/s. All spectra were acquired from 10° 2*θ* to 100° 2*θ*.

SEM analysis was performed using a LEO 1525 SEM and Zeiss Smart SEM software. For EDS analysis, the INCA software by Oxford Instruments was employed.

## Results and discussion

### Surface features

**Fig. 1:**
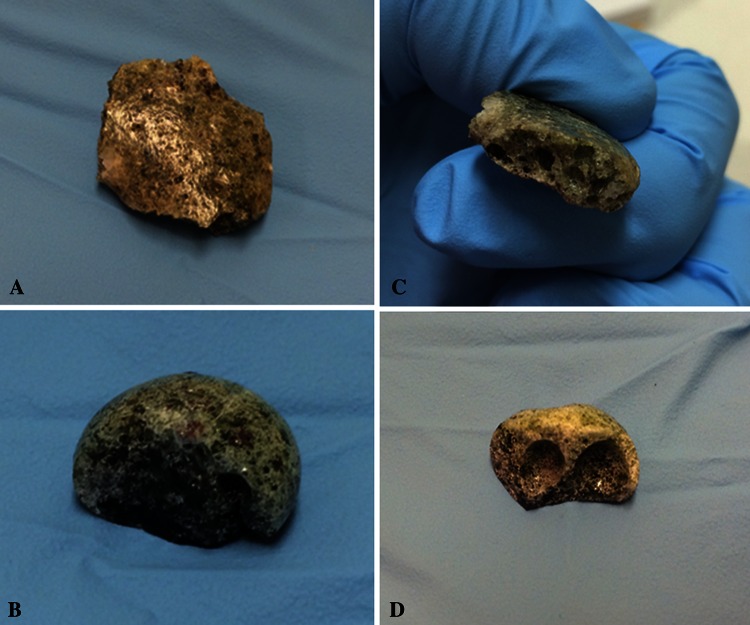
Photographs showing **a** Surface of a trinitite sample (T1), **b** Surface of a synthetic melt glass sample (S3), **c** Cross sectional view of a trinitite sample (T1), **d** Cross sectional view of a synthetic melt glass sample (S2)

By visual inspection of trinitite and synthetic melt glass many macroscopic similarities are immediately apparent, as seen in Fig. 1. The physical appearance of the surrogate debris is very similar to trinitite with a heterogeneous and vesicular appearance observable at the macroscopic level. These similarities are readily evident, as can be seen in Fig. 1. Image A shows the surface of sample T1. This can be compared with the surface of sample S3 shown in Fig. 1, Image B. Both surfaces are glassy with a greenish, brown color and apparent heterogeneity. The darker areas on the surface of the synthetic sample are believed to be carbon contamination from the graphite crucible. Point EDS analysis has provided evidence of carbon contamination in synthetic samples. This will be seen in the discussion regarding Fig. 4 in the crystallinity section. The obvious vesicular structure is perhaps the most noteworthy similarity between real and synthetic melt glass samples.

Figure 1, Image C shows a cross sectional view of sample T1 which can be compared to Image D which shows a similar view of sample S2. The vesicles seen in this particular synthetic sample are quite large, however, the vesicles observed in other synthetic samples had a wide range of sizes and shapes. In general the vesicles seen in synthetic samples appear to be somewhat larger, on average, but fewer in number than those seen in trinitite samples. The vesicular nature of the trinitite samples also varied widely. It is evident that the method employed produces synthetic samples with a structure similar to trinitite.

**Fig. 2:**
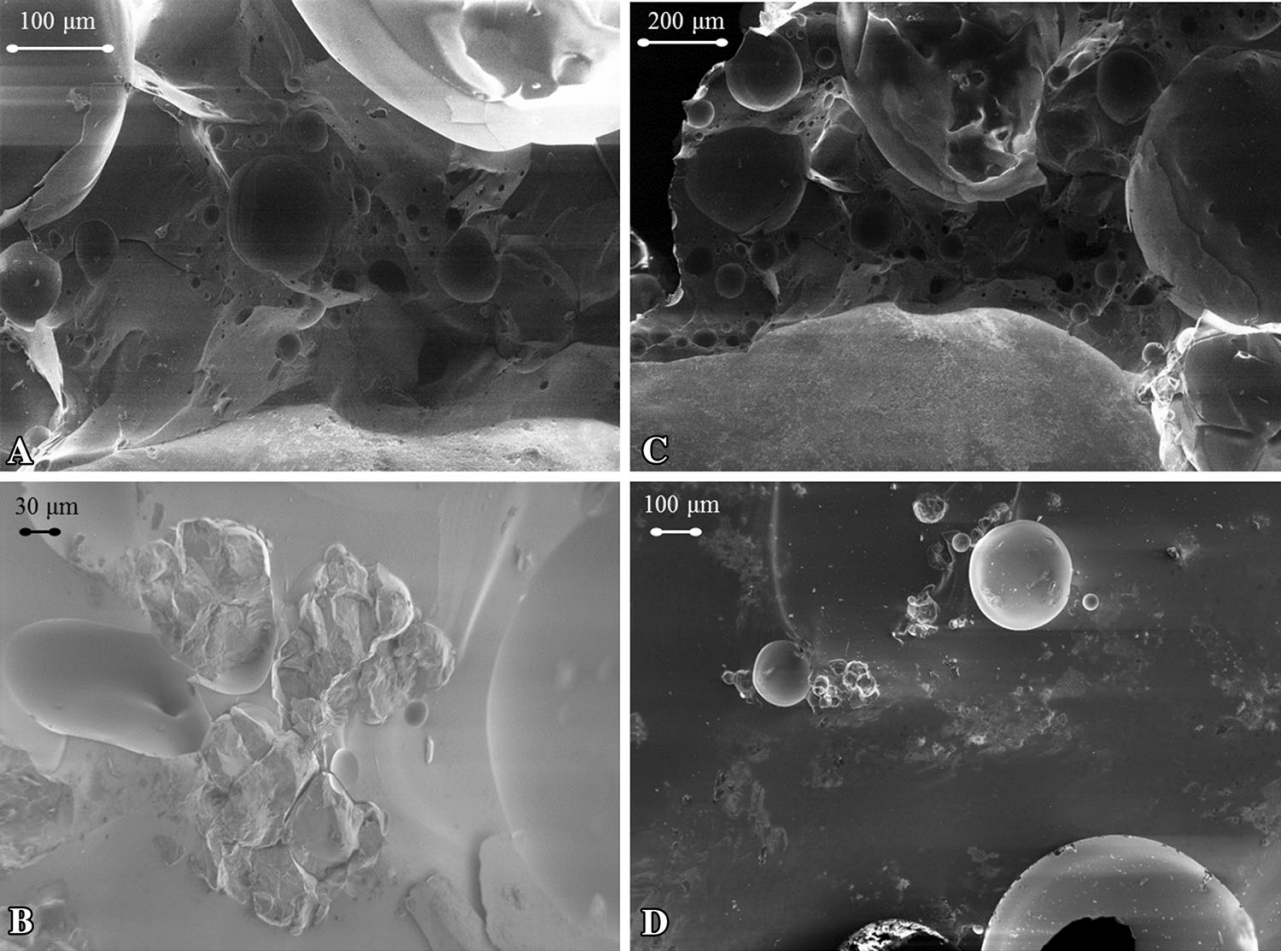
SEM micrographs of **a** Trinitite sample (T8) at 500 times magnification, **b** Synthetic melt glass sample (S4) at 500 times magnification, **c** Trinitite sample (T8) at 200 times magnification, and **d** Synthetic melt glass sample (S1) at 200 times magnification

The trinitite sample contains numerous vesicles of varied size along with cracks and other defects. The synthetic sample contains similar features, including a few large voids. At first glance Figs. 1 and 2 seem to suggest that trinitite may have a higher void content than the synthetic samples. However, the method described in the next section, used to estimate the void content of the synthetic samples, suggests otherwise.

Figure 2 compares two synthetic nuclear melt glass fragments to a trinitite fragment of similar size. Images A and B compare samples T8 and S4 at the same scale and reveal some similarities, including a few large vesicles and a varied texture in both samples. Sample T8 appears to contain a larger number of smaller voids, compared to sample S4. Images C and D compare samples T8 and S1 at the same scale. These images also reveal similarities in structure, void content, and texture. Images A and C in Fig. 2 depict different regions in a fragment of sample T8. It is evident that structure and texture may vary greatly, even within the same trinitite sample. Figures 1 and 2 together show that synthetic melt glass and trinitite samples have many similarities which are evident at magnifications ranging from 0 to 500 times.

Images B and D in Fig. 2 depict synthetic samples with slightly different processing parameters (see Table 2). Based on the observations, a general procedure to synthesize the samples includes a short exposure (45–60 min) at a high temperature (1,400–1,500 °C), which seems to produce the most realistic results for the trinitite (STF) matrix. However, these parameters depend rather sensitively on the composition of the samples. It has also been observed that void formation within the synthetic samples is unpredictable and surviving mineral content provides a better means of comparing trinitite and synthetic melt glass. This will be discussed further in the section on “Crystallinity”.

It should be noted here that features similar to those revealed in Fig. 2 have also been observed in nuclear melt glass from underground testing [14, 15].

### Void content

Previous studies have estimated the void content of trinitite to be approximately 30 % [10]. It is not clear to the authors how accurate the resin void volume experiment was at determining the volume. However, it should be noted that trinitite exhibits a high degree of variable from sample to sample in terms of void content and structure.

The void content of synthetic nuclear melt glass has not yet been quantified but is observed to vary greatly between samples, as is the case for trinitite. Furthermore, the voids and cavities in the trinitite are not connected by vesicular pathways, which will prohibit the authors from interrogating the void space with Brunauer–Emmett–Teller (BET) analysis. To gain an approximate volume of the voids in S1–S4, the mass of each oxide was measured and the final mass of the solid after the melt was recorded. The volume of the mixed STF compound was measured using standard water displacement, involving submerging a known mass of mixture in water, prior to melting, and measuring the volume change. Likewise, the final samples were submerged in water and the volume displacement was then recorded. Differences in density from the weighted average calculations and the measured density are recorded in Table 3, where ρ_mix_ is the density of the STF formulation prior to melting and ρ_heat_ is the measured density of the samples after heating. The void volume was obtained using Eq. 1:

**Table 3:** Comparison of the calculated and measured densities of synthetic trinitite

Sample	ρ_calc_ (g/mL)	ρ_heat_ (g/mL)	V_frac_ (%)
S1	1.81	1.02	39.8
S2	1.81	1.06	41.4
S3	1.81	1.07	41.4
S4	1.81	1.09	44.2

The average void volume (%) was determined to be 41.7 ± 1.8 %

1 $$ V_{\text{frac}} = \frac{{V_{v} }}{{V_{s} }} = 1 - \frac{{\rho_{\text{heat}} }}{{\rho_{\text{mix}} }}, $$

where *V*_frac_ is the void volume, *V*_*v*_ is the volume of the void in the melted mixture, and *V*_*s*_ is the volume of the melted solid. The results are given in Table 3.

### Composition

EDS analysis was performed to estimate the elemental content of a trinitite sample and a synthetic melt glass sample. The elemental data for these samples is consolidated in Table 4.

**Table 4:** EDS elemental composition data for samples T3 and S4 compared to trinitite composition from the literature

Element	Approximate weight fractions		
	Trinitite data [8]	Trinitite (T3)	STF glass (S4)
Si	3.00 × 10^−1^	2.18 × 10^−1^	2.69 × 10^−1^
Al	7.55 × 10^−1^	4.63 × 10^−2^	8.50 × 10^−2^
Ca	6.88 × 10^−2^	3.70 × 10^−2^	7.49 × 10^−2^
K	4.62 × 10^−2^	1.46 × 10^−2^	3.94 × 10^−2^
Na	9.23 × 10^−3^	n. d.	1.00 × 10^−2^
Fe	1.53 × 10^−2^	n. d	1.64 × 10^−2^
Mg	6.90 × 10^−3^	n. d.	6.2 × 10^−3^
Ti	2.58 × 10^−3^	n. d.	3.1 × 10^−3^
O	4.60 × 10^−1^	6.85 × 10^−1^	4.96 × 10^−1^

The elemental composition on the surface may not be representative of the entire sample. Because melting occurs in open air the chemistry of the sample may change due to high temperature reactions with oxygen and nitrogen in the atmosphere as well as carbon from the crucible and the atmosphere. Compounds with the highest oxygen content may be concentrated near the surface of the sample. Preliminary EDS mapping reveals a non-uniform elemental distribution in both trinitite and synthetic melt glass fragments. Data in Table 4 is thus only semi-quantitative and is shown primarily to demonstrate the sensitivity of both real and synthetic samples to EDS analysis. The discrepancies are likely due to the fact that development of the STF was based on averaged trinitite data and the exact composition of individual trinitite samples may vary significantly.

The elemental concentrations listed in the second column of Table 4 were based on chemical analysis of glassy regions within a trinitite fragment as well as several trinitite beads [8]. The STF composition was based on an average of these published data points.

### Crystallinity

P-XRD analysis shows that the trinitite was largely amorphous with the exception of a few peaks which were predominantly matched with quartz. Patterns were matched using the search and match function within the Panalytical analysis software.

The synthetic samples also contain quartz. The number and intensity of the observed peaks appears to depend rather sensitively on the melt time and temperature. This phenomenon will be discussed in a subsequent section.

**Fig. 3:**
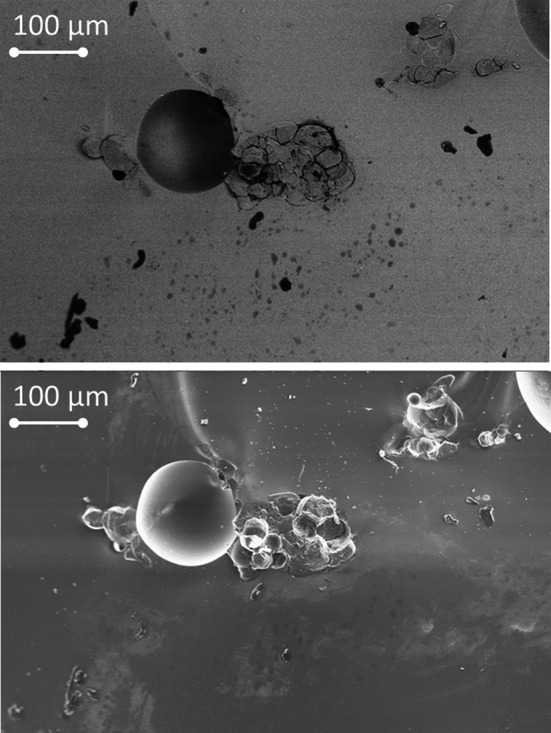
BSE (*top*) and SE (*bottom*) images of the same area on the surface of a fragmented synthetic melt glass sample. Both micrographs were captured at 408 times magnification

The two images shown in Fig. 3 are of the same feature on a fragment of synthetic melt glass. The secondary electron (SE) image shows only topographical features while the BSE image also shows relative differences in atomic number. The dark spots in the BSE image are low-Z grain inclusions within the glassy matrix. Point EDS has revealed that several of these inclusions are carbon debris particles picked up from the graphite crucible in which the sample was melted. At least one grain proved to be predominantly silicon—presumably a partially melted quartz inclusion. Similar inclusions have been found in trinitite samples [6, 8, 16]. Partially melted or un-melted quartz grains are a possible source of crystalline peaks which appear in the P-XRD patterns of both trinitite and synthetic melt glass samples.

**Fig. 4:**
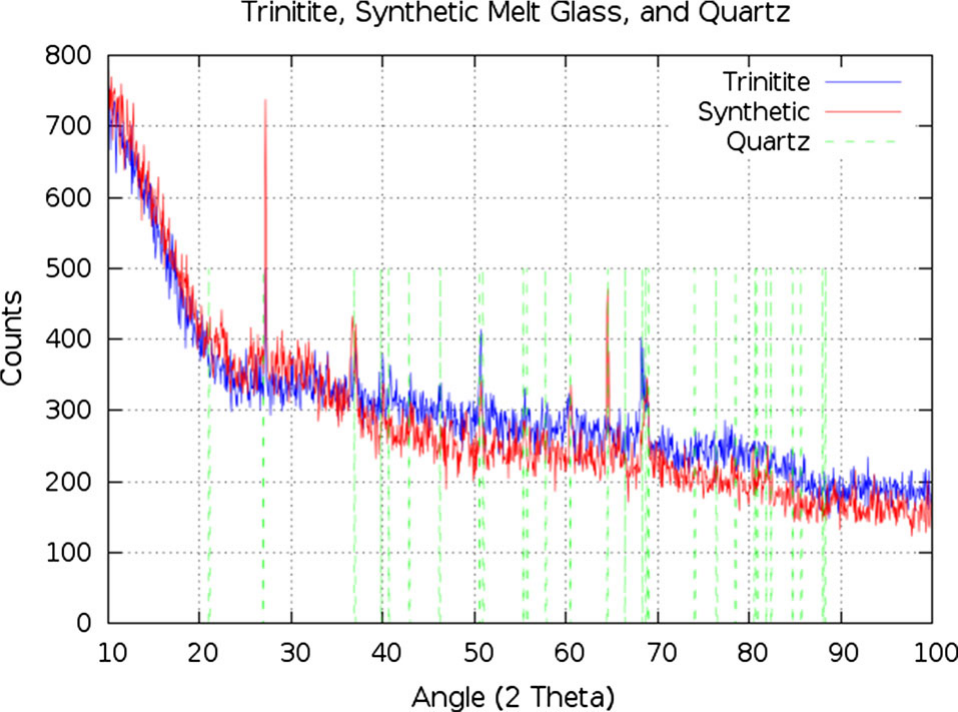
The sample T8 P-XRD pattern (*blue*) compared to the sample S2 P-XRD pattern (*red*) with *dashed green lines* showing the location of peaks in a typical quartz pattern

Figure 4 compares P-XRD patterns for trinitite (sample T8) and synthetic melt glass (sample S2). The locations of peaks in a typical quartz P-XRD pattern are also shown for comparison. While only a few of the quartz bands are observed in the trinitite P-XRD patterns, it is important to note that every peak which is observed corresponds to a quartz band. The same is true for the synthetic samples. In fact, the trinitite and synthetic peaks overlap quite nicely. And although the intensity of the individual peaks varies, the overall fraction of crystalline mineral content is comparable for both samples. For example, the trinitite pattern has strong peaks at 50° and 68° 2*θ*, while the strongest peaks in the synthetic pattern occur at approximately 27° and 64° 2*θ*, but the count totals are essentially the same.

In an effort to quantify the amorphous character of a sample the individual P-XRD peaks may be analyzed and compared to the background. By summing the counts under the prominent peaks and dividing by the total number of counts an approximate percentage can be assigned to the crystallinity of the sample. This analysis was performed on the patterns shown in Fig. 4, and it is estimated that the trinitite is 5.7 % crystalline and 94.3 % amorphous while the synthetic melt glass is 6.8 % crystalline and 93.2 % amorphous. Table 5 lists the results of this calculation for several trinitite and synthetic samples. In terms of parameter optimization, the data in Table 5 suggests that a lower limit exists at approximately 1,400 ^ο^C for 60 min, and an upper limit exists at approximately 1,500 ^ο^C for 45 min. Future experiments will focus on a precise optimization which also accounts for a comparable void content and structure.

**Table 5:** Trinitite and synthetic melt glass samples listed in order of percent vitrification

Sample	% Vitrified	Melt temp/time (^ο^C/min)
S2	93.18	1,400/45
T8	94.27	N/A
S1	94.52	1,400/60
T6	95.05	N/A
T3	97.05	N/A
S4	98.57	1,500/45
S3	99.35	1,500/60

### Effect of melting temperature on morphology and crystallinity

**Fig. 5:**
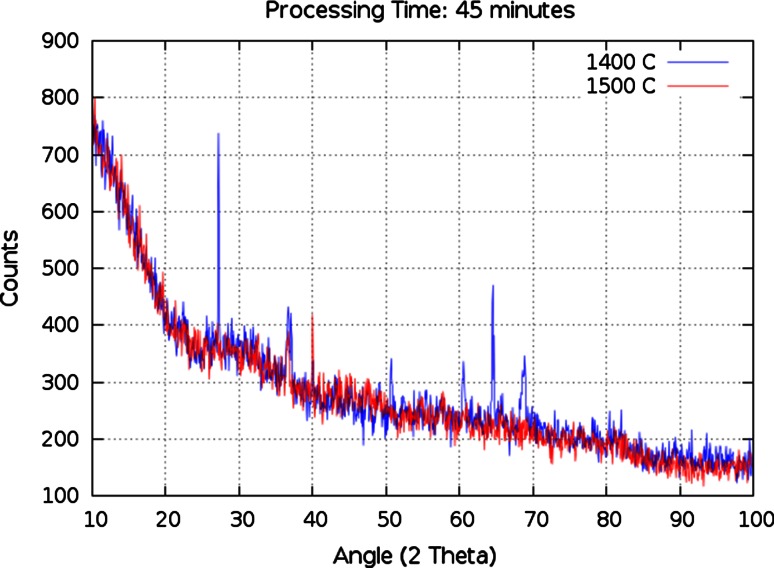
P-XRD crystalline peak comparison of samples S2 (*blue*) and S4 (*red*)

It is well documented that quartz is generally the only mineral that survives in trinitite [8, 9]. It is desirable that surrogate melt glass exhibit a similar degree of amorphousness. The processing parameters which have the greatest impact on the amorphous or crystalline nature of the melt glass are temperature, melt time and cooling rate. Figure 5 compares two synthetic samples with the same composition and identical processing parameters, with the exception of the melting temperature. Both samples were melted for 45 min in graphite crucibles. A 100 °C increase in melting temperature significantly reduces the number and intensity of crystalline peaks identified via P-XRD analysis.

### Effect of melting time on morphology and crystallinity

**Fig. 6:**
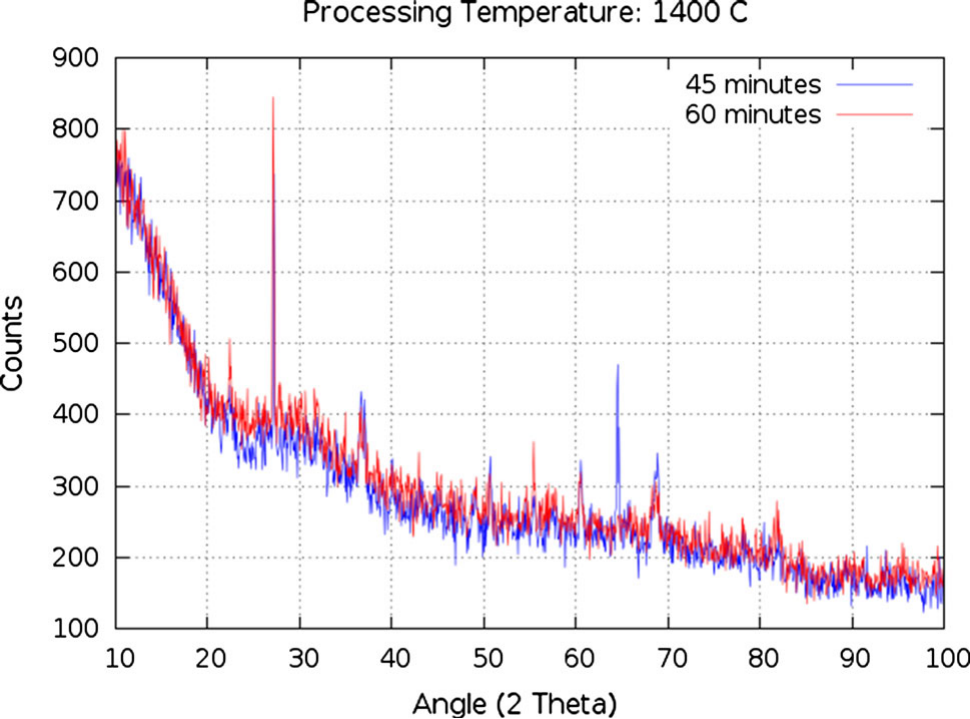
P-XRD Crystalline peak comparison of samples S1 (*red*) and S2 (*blue*)

Figure 6 compares two surrogates with the same composition, which were both melted at 1,400 °C, but for different durations. The X-ray spectra are very similar with the exception of a prominent peak at 65° 2*θ* which is seen only in the 45 min melt data. This peak is easily associated with quartz, as are the remainder of the peaks seen in both spectra. The longer melt retains some quartz but with fewer prominent peaks. This comparison demonstrates that a 25 % increase in melting time produces a noticeable decrease in crystallinity.

### Variability within trinitite

**Fig. 7:**
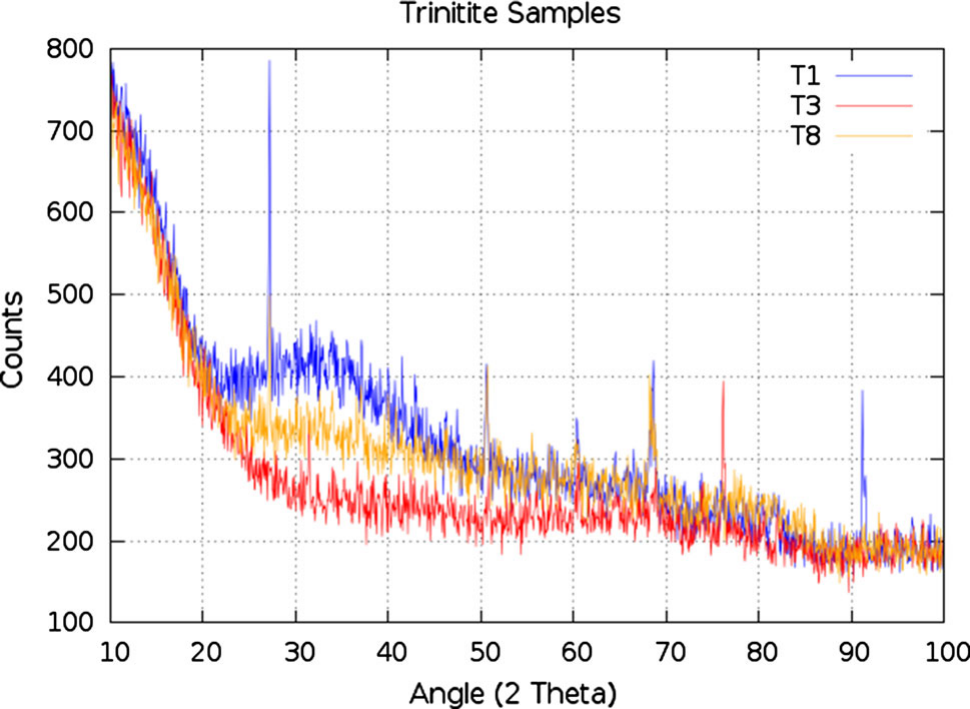
Comparison of P-XRD patterns for three different trinitite samples

It is noteworthy that the P-XRD data compiled on three different trinitite samples show some variability in terms of crystallinity and vitreous nature, as shown in Fig. 7.

While the original location of these samples within the trinitite debris field is unknown, it can be shown that melt glass near the outer edge (farthest away from ground zero) may be subjected to temperatures as low as 1,400 °C.

Heat radiated from the epicenter of a nuclear detonation is governed by Eq. 2 [17].

2 $$ Q = \frac{{Yf\tau (10^{12} )}}{{4\pi D^{2} }} \;{\text{cal/cm}}^{2} , $$

Here *Y* is the yield of the weapon, *f* is the thermal partition (a parameter unique to each explosion which depends on yield and height of burst), and *τ* is the transmittance (fraction of thermal radiation transmitted). For surface and tower bursts a typical value for the thermal partition is *f* = 0.35. On a clear day an approximate transmittance value for a surface burst is *τ* = 0.9 [17]. Using the specific heat and density of silicon dioxide, the radiated heat can be converted to a temperature (in °C).

**Fig. 8:**
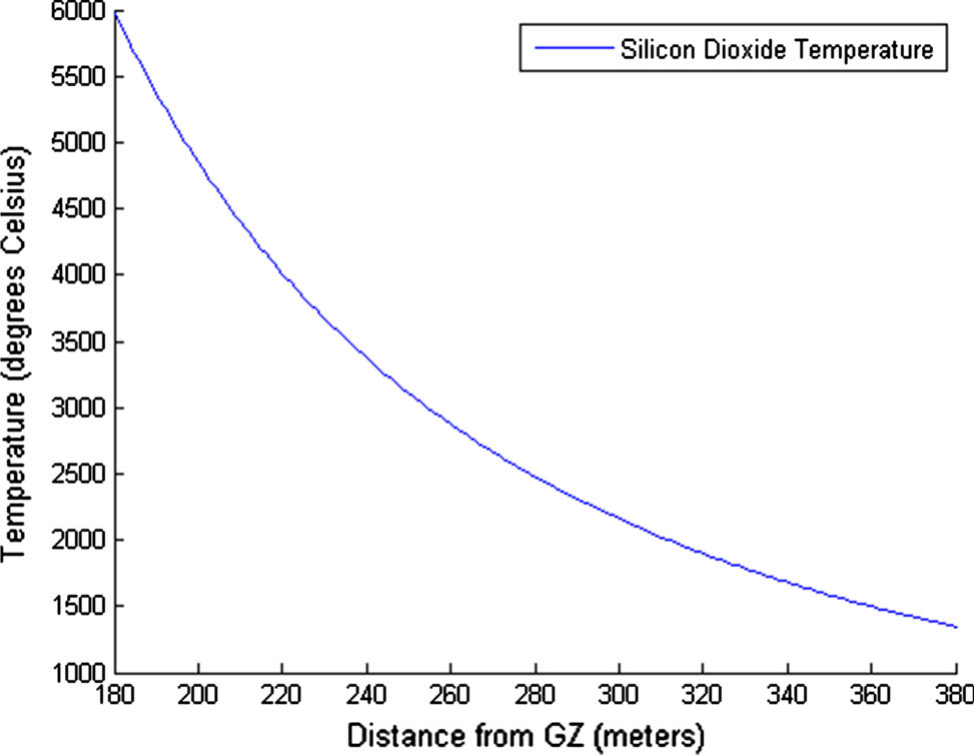
Temperature of silicon dioxide as a function of distance from ground zero (GZ) induced by a 21 KT nuclear detonation

Figure 8 shows the estimated temperature to which SiO_2_ is raised as a function of distance from ground zero. This could explain why quartz survives in trinitite to varying degrees. The temperatures estimated using Eq. 2 are applicable to melt glass formed directly on the ground. The temperature distribution within the rising fireball may be quite different. However, debris that is drawn into the fireball will only be superheated for 2–3 s. This short exposure time may allow some particles to escape un-melted or only partially melted. Irregular winds within the cloud may also have an impact on temperature and dwell time. The fact that nuclear melt glass contains a mixture of fused sand (which was melted directly on the ground) and droplets from the cloud will likely enhance its variability in terms of morphology and crystallinity.

It is important to emphasize that the high degree of variability within trinitite samples suggests that similar variability within synthetic melt glass is acceptable, provided that quartz is the only prevalent mineral phase. The samples produced for this study are consistent with this observation.

It is worth noting that the P-XRD pattern for one trinitite sample (red line in Fig. 7) includes a peak (at ~31.5° 2*θ*) which does not match with quartz. The source of this peak has not yet been conclusively identified, however, at least one instance of dendritic iron crystallization has been observed previously in trinitite [16]. Preliminary analysis suggests that the outlier peak observed in Fig. 7 may result from a crystalline form of a lead or magnesium compound.

### Effect of cooling rate on morphology and crystallinity

The sample cooling rate after removal from the furnace was difficult to quantify. Ideally, the glass would be poured and quenched in sand at room temperature, however, samples sometimes became fused inside the crucible. When pouring was not possible the glass bead would be allowed to cool just long enough to solidify (about 60 s) and then removed from the crucible with a pair of tongs. Graphite retains heat for a considerably period of time so it is desirable to remove the glass as soon as possible to avoid recrystallization due to slow cooling rates.

## Conclusions

A method for producing synthetic nuclear melt glass has been tested and the samples characterized using P-XRD, SEM, and EDS. The starting formulation for the synthetic samples was based on published data partially quantifying the oxide composition of trinitite (the original and most readily available form of nuclear melt glass). It has been shown that a reasonable set of processing parameters can be employed to produce synthetic melt glass with physical, chemical, and morphological properties very similar to trinitite. In particular, a high degree of vitrification can be achieved at temperatures between 1,400 and 1,500 °C with melt times between 30 and 60 min.

Additional analysis will be required to optimize the production process. It is clear that, from a physical and morphological standpoint, a realistic surrogate is obtainable. Optical, morphological and chemical data collected on trinitite and synthetic melt class proved to be comparable.
